# Recurrent Jejunojejunal Intussusception of the Common Limb in a Patient With Roux-en-Y Gastric Bypass: A Report of an Unusual Case and Literature Review

**DOI:** 10.7759/cureus.109400

**Published:** 2026-05-21

**Authors:** Andrea Navalón Calzada, Oscar Gerardo Chavéz Cadena, Begoña Reyes Rementeria, Roberto Turrent Pinedo

**Affiliations:** 1 Department of General Surgery, Spanish Hospital, Mexico City, MEX

**Keywords:** common limb, intestinal obstruction, intussusception, jejuno-jejunal intussusception, roux-en-y gastric bypass (rygb)

## Abstract

Intestinal intussusception is a rare late complication of Roux-en-Y gastric bypass (RYGB), typically presenting without a lead point, often in a retrograde pattern, and most commonly involving the jejunojejunal anastomosis and common limb. We report the case of a 49-year-old female with a history of RYGB performed in 2000 and prior intussusception in 2010, who presented with an acute abdomen. Contrast-enhanced computed tomography demonstrated intestinal obstruction secondary to recurrent jejunojejunal intussusception, with associated signs of vascular compromise. The patient underwent exploratory laparotomy with successful manual reduction of an approximately 50 cm segment of the common limb, resulting in a favorable postoperative course. Although optimal surgical management remains debated, approaches combining reduction with fixation and revision of the jejunojejunal anastomosis may reduce the risk of recurrence. This case highlights the importance of maintaining a high index of suspicion for prompt diagnosis and management of this uncommon but significant late complication.

## Introduction

Intestinal intussusception in adults is an uncommon clinical entity, accounting for approximately 1-5% of all cases of bowel obstruction and less than 5% of all intussusceptions [1,2]. Unlike pediatric cases, it is often associated with a pathological lead point such as neoplasms, postoperative adhesions, or structural abnormalities [2]. Particularly, in patients with prior Roux-en-Y gastric bypass (RYGB), intussusception represents a rare but increasingly recognized late complication, with an estimated incidence ranging from 0.1% to 0.3% [3,4].

The pathophysiology of post-RYGB intussusception differs from the classic mechanism, as it is frequently characterized by a retrograde pattern and occurs in the absence of a clear lead point [4,5]. Briefly, RYGB anatomy consists of a gastric pouch anastomosed to the Roux (alimentary) limb, which carries food, and a biliopancreatic limb that transports digestive secretions; these two limbs join at the jejunojejunal anastomosis to form the common channel [3]. Proposed mechanisms include dysmotility related to disruption of normal intestinal pacemaker activity, particularly involving the jejunal limb, as well as alterations in peristaltic coordination following surgical reconstruction [3,5]. This atypical presentation often involves the jejunojejunal anastomosis and the common channel, making diagnosis particularly challenging [3].

Clinically, patients may present with nonspecific symptoms such as intermittent abdominal pain, nausea, vomiting, or signs of acute bowel obstruction [2,6]. Imaging studies, particularly contrast-enhanced computed tomography, play a crucial role in diagnosis, with characteristic findings including the “target” or “sausage-shaped” sign [7]. Given its rarity and nonspecific presentation, a high index of suspicion is required, especially in patients with a history of bariatric surgery presenting with an acute abdomen.

Surgical management remains controversial, with options ranging from simple reduction to resection and revision of the jejunojejunal anastomosis, with or without enteropexy [3,4]. Recurrence rates vary depending on the chosen approach, and no consensus has been established regarding the optimal surgical strategy.

Despite the growing number of bariatric procedures performed worldwide, recurrent intussusception after RYGB remains an exceptionally rare condition, with limited cases reported in the literature [3,6]. Therefore, this case report aims to contribute to the existing evidence by describing an unusual presentation of recurrent jejunojejunal intussusception, highlighting its clinical, radiological, and surgical features, and emphasizing the importance of early recognition and appropriate management to prevent severe complications such as bowel ischemia and necrosis.

## Case presentation

A 49-year-old female patient was admitted to the emergency department presenting with an acute abdomen. Her surgical history included an elective RYGB performed in 2000 for class II obesity with a BMI of 36 kg/m^2^ . In 2010, she experienced a previous episode of intestinal obstruction due to jejunojejunal intussusception with associated bowel ischemia. This was initially managed via laparoscopic reduction; however, a second intervention was required due to recurrent intussusception. During the subsequent procedure, a bowel resection of the affected segment was performed, followed by primary anastomosis and reconstruction of the Roux-en-Y limb.

Clinical presentation

The patient began her current condition with sudden onset of diffuse, sharp abdominal pain, with an initial intensity of 10/10 on the Visual Analog Scale (VAS), exacerbated in the supine position. This was accompanied by two Bristol type 4 bowel movements, without mucus or blood, providing partial pain relief. Due to the persistence of symptoms, she sought emergency care. Physical examination revealed tachycardia (105 bpm) and hypertension (162/85 mmHg); other vital signs were within normal parameters. The abdomen was distended and rigid, showing clear signs of peritoneal irritation with positive rebound tenderness and involuntary guarding. It was firm and poorly compressible upon palpation. Auscultation revealed high-pitched "tinkling" bowel sounds (struggle sounds), and percussion was hypertympanic.

Diagnostic studies

Initial laboratory studies showed leukocytosis of 13.5 × 103/µL with neutrophilia (76.7%) and 10% bands, glucose of 126 mg/dL, and creatinine of 1.05 mg/dL. Plain abdominal radiography evidenced a decrease in the colonic gas pattern with scarce residue (Figure 1).

**Figure 1:**
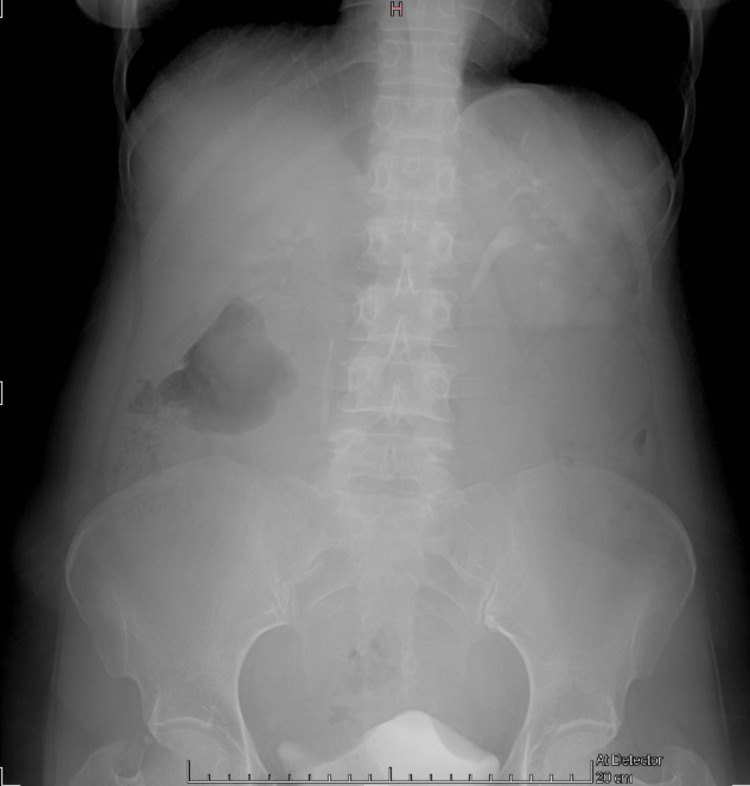
Abdominal X-ray demonstrating a colon with decreased habitual gas pattern; caliber is preserved with scarce amount of residue.

Contrast-enhanced abdominal computed tomography documented intestinal obstruction secondary to intussusception of a long segment of jejunum, with signs of bowel compromise and postoperative changes compatible with RYGB (Figures 2-4).

**Figure 2:**
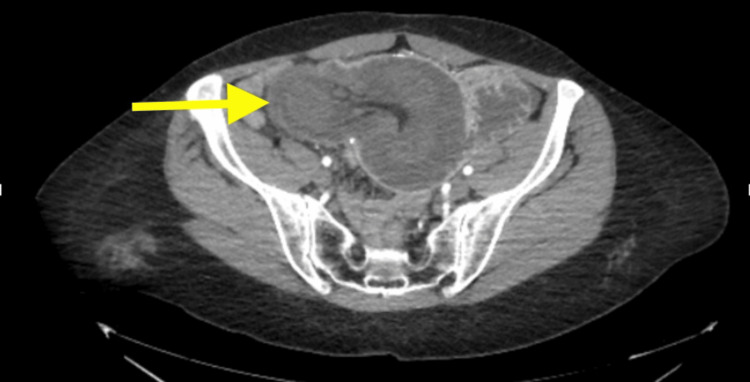
Contrast-enhanced abdominal computed tomography (CT), axial view: small intestine at the jejunal level demonstrating a telescoped intestinal segment within another, producing the characteristic target sign (arrow).

**Figure 3:**
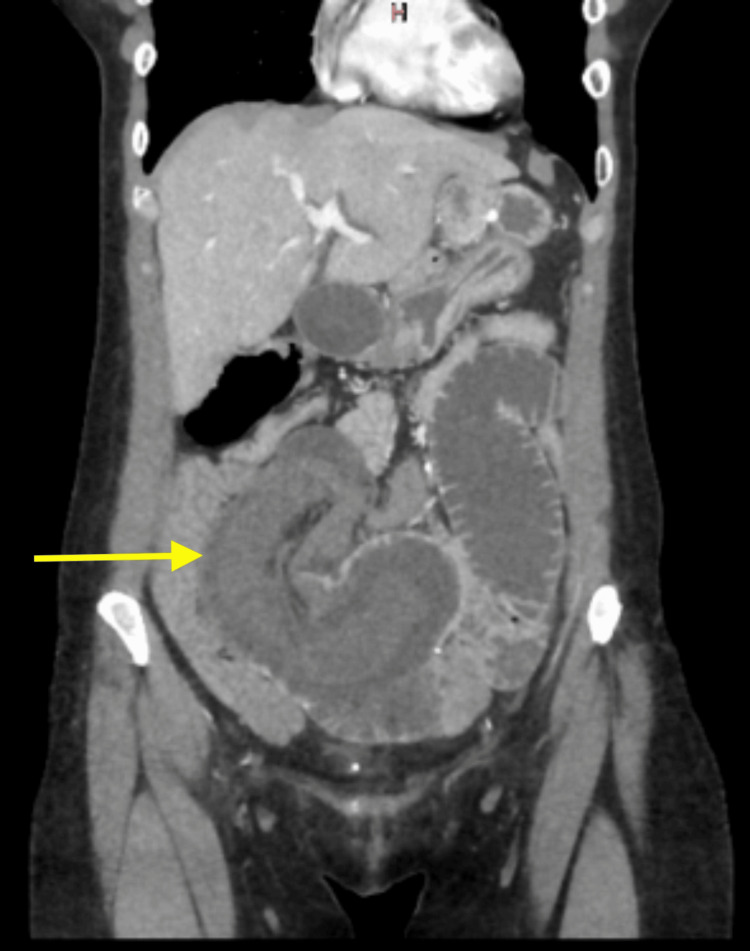
Contrast-enhanced abdominal tomography, coronal view: the intussuscepted segment demonstrates concentric bowel wall thickening, mural edema, and decreased mural attenuation consistent with vascular compromise, associated with marked proximal small bowel dilatation (arrow).

**Figure 4:**
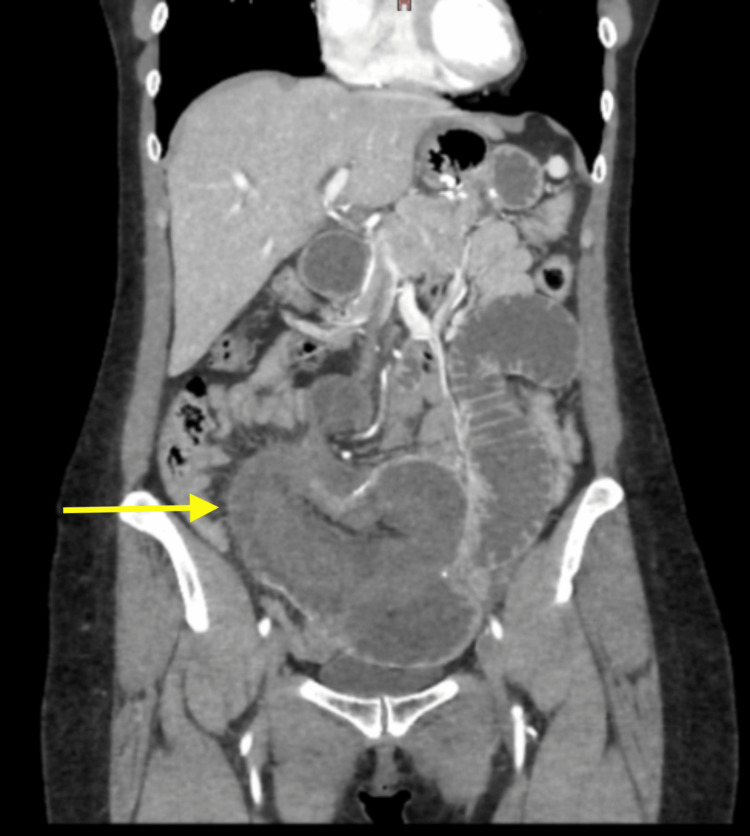
Contrast-enhanced abdominal tomography, coronal view: invaginated mesenteric fat and mesenteric vessels are identified within the intussuscepted bowel segment, producing the characteristic bowel-within-bowel appearance (arrow).

Based on these findings, a diagnosis of intestinal obstruction secondary to jejunojejunal intussusception with common limb involvement was established, and urgent surgical management was indicated.

Surgical management

An exploratory laparotomy was performed via a midline supra- and infraumbilical incision. Markedly dilated jejunal loops and the previous jejunojejunal anastomosis were identified, revealing an intussusception of the common limb with discoloration suggestive of ischemic compromise. The intussusception involved approximately 50 cm of jejunum, located 60 cm from the ligament of Treitz. Manual reduction was successfully performed. Following irrigation with warm saline, bowel viability and peristalsis were confirmed. To minimize the risk of recurrence, a triple pexy (fixation) was performed by placing anchoring sutures between the common limb, the alimentary limb, and the biliary limb. Additionally, due to significant dilation, a decompression tube was placed in the biliary limb. The procedure was completed with the placement of a tunneled feeding jejunostomy and a Jackson-Pratt drain in the left paracolic gutter.

Postoperative course

The patient demonstrated a favorable postoperative course, with mild incisional pain (3/10 on the Numeric Rating Scale (NRS)) controlled with analgesics. The drain and nasogastric tube initially showed minimal hematic output, with progressive improvement. Given the absence of emesis and the reestablishment of peristalsis, the nasogastric tube was removed, and enteral nutrition was initiated with adequate tolerance, passage of flatus, and bowel movements.

## Discussion

Intestinal intussusception in adults is an infrequent entity, accounting for less than 5% of intussusception cases and approximately 1-5% of intestinal obstruction causes in this age group [1]. In most cases, it is associated with an identifiable lead point, such as benign or malignant tumors, polyps, or inflammatory lesions [1,2]. However, in patients with a history of RYGB, intussusception represents a rare late complication, with a reported incidence between 0.1% and 0.3%, presenting distinct clinical, anatomical, and pathophysiological characteristics compared to the general population [3-6]. In this context, intussusception usually presents without a lead point, with a predominantly retrograde pattern and preferential localization at the jejunojejunal anastomosis, frequently involving the common limb [3-8]. Timely recognition is crucial, as the clinical presentation is often nonspecific and can rapidly progress to intestinal ischemia, necrosis, and sepsis if diagnosis or treatment is delayed [4,6].

Post-RYGB intussusception constitutes a clinical entity distinct from intussusception in the non-operated adult. Various reviews and series agree that the predominant localization is the jejunojejunal anastomosis, with frequent involvement of the common limb and a retrograde pattern [3-8]. This behavior contrasts with the typical antegrade intussusception of the pediatric population and adult intussusception associated with organic lesions [1,2].

The case presented by Samad et al. [6] is particularly illustrative, describing an extensive retrograde intussusception (≈120 cm) of the distal common limb into the jejunojejunal anastomosis. Despite an initially successful reduction, the patient had an unfavorable evolution, developing sepsis and intestinal necrosis that required extensive resection and reconstruction of the anastomosis. This clinical behavior supports the notion that common limb involvement can be extensive and dynamic and carries a risk of delayed ischemia, justifying a low threshold for surgical re-exploration in the face of clinical deterioration [6]. Epidemiologically, consistent patterns have been identified: female predominance, late presentation, and association with significant weight loss [3-8].

Regarding pathophysiology, the main mechanism is considered functional rather than structural. Jejunal transection during RYGB separates the Roux limb from the influence of the duodenal pacemaker, favoring the appearance of ectopic electrical activity and abnormal motility patterns [9,10]. This phenomenon has been linked to the so-called "Roux stasis syndrome" and the generation of antiperistaltic waves capable of producing retrograde intussusception [8-10]. Hocking et al. described this association between dysmotility and intestinal intussusception in Roux reconstructions early on [8].

Anatomically, the jejunojejunal anastomosis acts as a vulnerable point where changes in caliber, angulation, and staple lines converge, facilitating intestinal invagination when anomalous motility forces coexist [3-7]. In this context, the common limb appears to be particularly susceptible, either as the intussuscepted segment or as part of the jejunojejunal anastomosis-common limb complex, as observed in both the present case and multiple reports in the literature [3-8].

Diagnosis relies fundamentally on contrast-enhanced computed tomography, which allows identification of the "target" or "sausage" sign, definition of the location at the jejunojejunal anastomosis or common limb, and detection of signs of obstruction or bowel compromise [4,7].

Regarding treatment, there is no definitive consensus; however, most authors recommend early surgical exploration in the presence of obstruction, severe pain, or suspicion of ischemia [3,4,7]. Described strategies include simple reduction, reduction associated with enteropexy, and bowel resection with revision or reconstruction of the jejunojejunal anastomosis [4,5,7,11]. Available evidence suggests that while revision of the jejunojejunal anastomosis and reduction associated with enteropexy present the lowest recurrence rates, isolated reduction is associated with significantly higher recurrence rates [6,11]. This is vividly illustrated by our index patient, who experienced multiple recurrences following both simple reduction and subsequent anatomical refashioning, achieving stability only after a triple pexy was performed. This highlights that no single modality is entirely efficacious on its own; therefore, we advocate that pexy should be combined with initial reduction or refashioning as a primary strategy to further reduce the likelihood of recurrence.

Furthermore, given the significant morbidity and high recurrence rates demonstrated in our index patient, a broader preventative question arises regarding whether an enteropexy should be routinely performed during the initial RYGB procedure. While a prophylactic pexy at the time of the primary bariatric surgery could theoretically eliminate the mobility of the jejunal limbs and prevent future retrograde intussusception, current evidence remains limited. Routine fixation could potentially introduce new technical challenges, such as creating areas for internal herniation or kinking [12]. Ultimately, while routine prophylactic pexy remains a subject of debate, this case underscores that surgeons should maintain a low threshold for incorporating stabilizing maneuvers when high-risk anatomical or functional predisposing factors are identified.

## Conclusions

Jejunojejunal intussusception with common limb involvement constitutes an infrequent but distinctive late complication of RYGB, characterized by a retrograde pattern and a predominantly functional pathophysiology rather than the presence of a structural lead point. Its nonspecific clinical presentation frequently leads to diagnostic delays, increasing the risk of progression to intestinal ischemia, necrosis, and septic complications.
